# Rehabilitation nursing for brain tumor patients: a scoping review

**DOI:** 10.1186/s12909-025-07423-0

**Published:** 2025-06-02

**Authors:** Runa Tokunaga, Miki Sakaki, Satomi Kashiwa, Naoko Hayashi

**Affiliations:** https://ror.org/00e5yzw53 grid.419588.9 0000 0001 0318 6320 St. Luke’s International University, 10-1, Akashi-cho, Chuo-ku, Tokyo, 104-0044 Japan

**Keywords:** Brain tumor, Brain neoplasms, Rehabilitation, Nurse, Review

## Abstract

**Objective:**

There is a lack of systematic reports on the status and roles of nurses in rehabilitation that significantly impact the functional prognosis and quality of life of brain tumor patients. Therefore, the purpose of this study was to conduct a scoping review on rehabilitation nursing for brain tumor patients.

**Methods:**

Using the JBI Manual for Evidence Synthesis, this research employed a scoping review design. CINAHL plus and PubMed databases were searched for literature published from 1947 to November 2023. Inclusion criteria covered articles including rehabilitation nursing for brain tumor patients and excluding pediatric studies, case reports, discussion papers, editorials, and expert opinions. The study is registered with UMIN-CTR (ID: UMIN000053136).

**Results:**

Following the adoption criteria, of the 2748 articles found 19 were selected. The literature selection adheres to PRISMA guidelines. The research designs included two RCTs, five longitudinal intervention studies, three systematic reviews, eight literature reviews, and one questionnaire survey. Among them, 15 articles discussed rehabilitation provided by nurses within a multidisciplinary rehabilitation, while four articles specifically addressed rehabilitation as one aspect of nursing care for brain tumor patients. The identified aspects were categorized as: information gathering and organization, meaning for the continuation of rehabilitation, prevention and management of secondary complications, family support, and collaboration with other healthcare professionals, all of which were consistently explained in all studies. Current challenges in rehabilitation nursing included a lack of studies on outcome measurement, insufficient knowledge and skills among nurses, and underdeveloped specialization in the field.

**Conclusions:**

Nurses play a crucial role in collecting patient information and collaborating with healthcare professionals for effective rehabilitation. They address symptoms arising from the disease and treatment, contributing significantly to rehabilitation’s effectiveness. However, research on rehabilitation nursing for brain tumor patients is limited, highlighting the need for further development in this area.

## Background

Brain tumors can occur in various parts of the brain parenchyma, resulting in a wide range of symptoms [1]. Previous studies targeting patients with such characteristics have reported numerous difficulties in daily life associated with symptoms, such as shock from loss of physical function and independence [2], facing the reality of not being able to return to their former selves [3], and confusion in patients’ lives [4]. Furthermore, advancements in treatment for brain tumor patients have extended their lifespan, implying a potential increase in the complexity of difficulties they face in daily life. Against this backdrop, the necessity for care aimed at maintaining functional independence has become increasingly critical [5]. 

Supportive therapy aimed at improving the quality of life of cancer patients is positioned as care to alleviate and prevent symptoms caused by tumors or treatments [6]. Among these, rehabilitation is performed with the aim of improving functional abilities and quality of life to the greatest extent possible for individuals facing life problems [7]. Therefore, this can be considered as one effective supportive therapy for brain tumor patients experiencing difficulties in daily life due to the impact of tumors or treatments.

In previous studies, rehabilitation for brain tumor patients has been reported to be effective in improving functional prognosis and quality of life [8], and proactive implementation is recommended [9]. While physicians and therapists play central roles in rehabilitation, comprehensive rehabilitation involving multidisciplinary collaboration, including nurses, has been shown to yield better outcomes [10]. Within this multidisciplinary collaboration, nurses are expected to connect care to patients’ lives by addressing issues such as patient adherence, continuous nursing care, alleviation of patient distress and stress, decision-making regarding lifestyle choices and subsequent home support, and reconstruction of meaningful lives [11, 12], all while addressing the impaired functions of brain tumor patients [13]. Thus, nurses play a significant role in rehabilitation for brain tumor patients within multidisciplinary collaboration. However, there is scarce integration of reports focusing on rehabilitation nursing for malignant brain tumor patients in previous studies.

Therefore, this study aimed to conduct a scoping review of nursing-led rehabilitation for brain tumor patients. This allowed for clarification of the realities of nursing in rehabilitation for brain tumor patients and the role of nurses. Additionally, by linking nursing’s daily life support with rehabilitation, it is believed that this study could serve as foundational material for improving the quality of care provided to brain tumor patients. This is expected to lead to the alleviation of confusion and distress in the lives of brain tumor patients with distinctive symptoms, ultimately contributing to maintaining independent living.

## Objective

To conduct a scoping review on the rehabilitation conducted by nurses for patients with brain tumors.

## Methods

### Research design

In this study, we systematically conducted a scoping review of the research findings. Following the framework proposed by Arksey & O’Malley [14] and developed by Levac et al. [15] and the Joanna Briggs Institute [16], we adhered to the guidelines outlined in the JBI Manual for Evidence Synthesis [17] to summarize and synthesize the research results.

### The extraction of literature

The databases used were CINAHL Plus and PubMed. The search period was not limited, with November 3, 2023, as the search date. We believe that conducting a comprehensive literature search without restricting the research period will allow for a thorough investigation and synthesis of evidence on rehabilitation nursing for brain tumor patients. All literature in English published from 1947 until that date was targeted.

The selection criteria for the literature are defined based on the following PCC (Population, Concept, and Context) for this review are as follow:

- Population (P): Nurses conductiong rehabilitation for glioma patients.
- Concept (C): Rehabilitation (rehabilitation, rehabilitation nursing).
- Context (C): Worldwide (no restriction on research location).

This review aims to address the following research questions:

- What is the practice of rehabilitation nursing for brain tumor patients?
- What is the scale for measuring the effectiveness of rehabilitation nursing for brain tumor patients?

The inclusion and exclusion criteria for this study are as follow:

- Inclusion criteria is (1) studies focusing on adult brain tumor patients, (2) studies mentioned in rehabilitation nursing.
- Exclusion criteria is (1) studies focusing on pediatric subjects, (2) studies with research design such as case report, discussion papers, commentaries, editorials, or expert opinions.

The search was conducted using the following keywords: (glioma or brain neoplasms) and (rehabilitation or rehabilitation nursing or nursing or nurses) in MeSH terms and title/abstract. Duplicate papers were removed during the screening process. By reviewing titles and abstracts according to the inclusion and exclusion criteria menthined above, articles were selected. All identified records were grouped, duplicates were removed, titles and abstracts were selected by three independent reviewers (●,●,●), and the full text of selected articles was evaluated in detail. Specifically, the full texts were carefully reviewed, and literature containing information on rehabilitation by nurses for brain tumor patients was selected, resulting in the extraction of 19 articles, which were included for analysis in this study. The flowchart for literature selection was created following the Preferred Reporting Items for Systematic Review and Meta-Analysis (PRISMA) guidelines [18]. 

During the selection and eligibility process, three researchers using the online platform Rayyan QCRI [19]. In cases of disagreement among researchers, discussions were held until consensus was reached, and irrelevant studies were excluded. This research protocol was registered with UMIN-CTR (ID: UMIN000053136).

### Analysis method

First, the extracted literature was organized by author, publication year, country of publication, objectives, research design, methods, interventions, scales used, and main results and discussions. Subsequently, a systematic analysis was conducted on the content and effectiveness of rehabilitation conducted by nurses for brain tumor patients.

## Ethical considerations

The handling of literature involved citing sources, ensuring no infringement of copyrights, and striving to faithfully reference the original papers as ethical considerations.

## Results

**Fig. 1:**
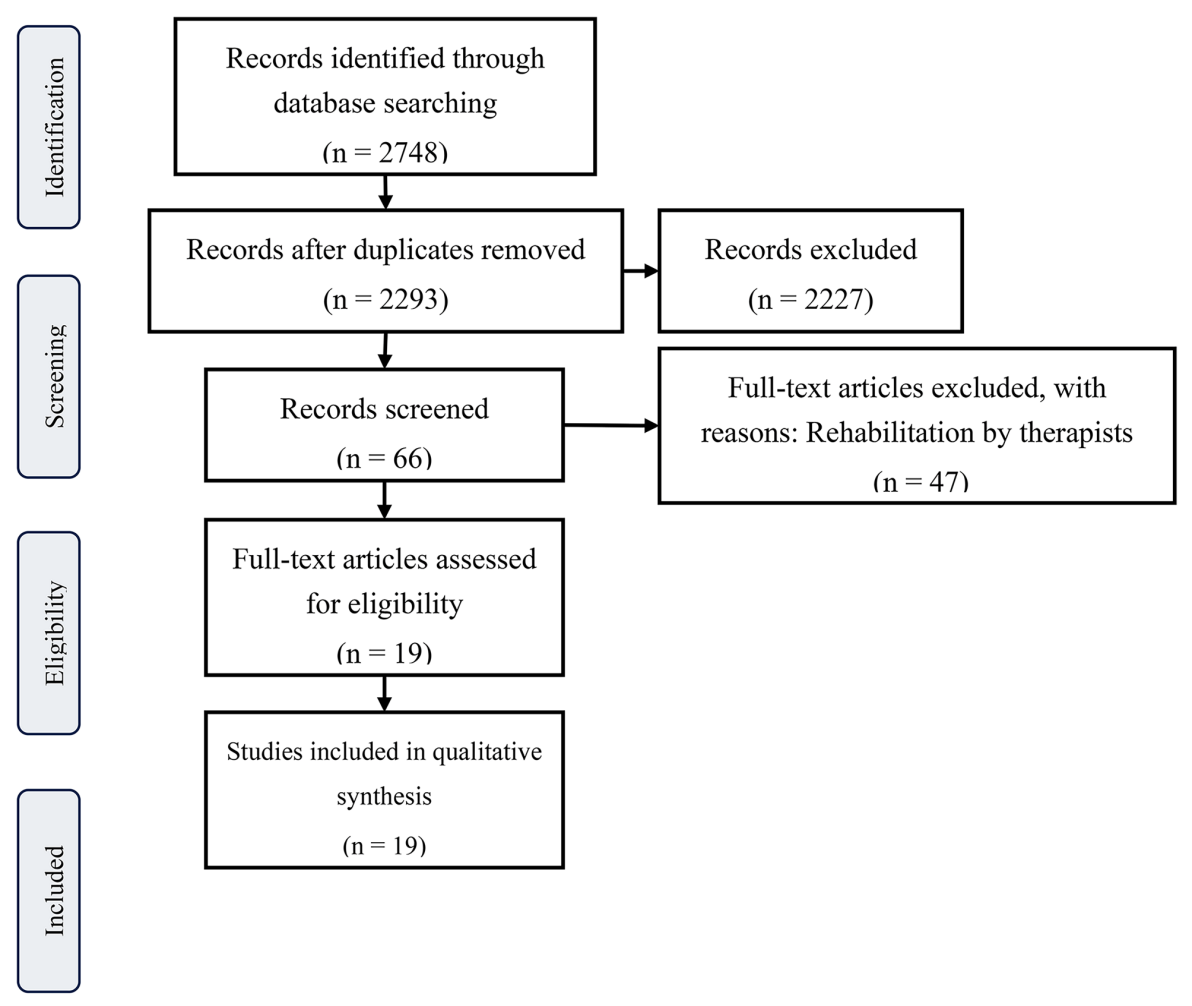
Flowchart for Literature Selection

Figure 1 illustrates the process of literature selection. A total of 2748 articles were retrieved from CINAHL Plus and PubMed. After excluding 455 duplicate articles, 2293 articles remained for primary screening. During the primary screening, 2227 articles were excluded after reviewing their titles and abstracts, leaving 66 articles for further examination. Subsequently, during the secondary screening, 47 articles were excluded after reviewing the full texts, resulting in a final selection of 19 articles for review. The summary of the included articles is presented in Table 1.

### Summary of the target literature

The 19 selected articles were organized by publication year as follows: one article published before the 2000s, one article published from 2000 to 2004, one article published from 2005 to 2009, four articles published from 2010 to 2014, four articles published from 2015 to 2019, and eight articles published from 2020 to 2023. In terms of study design, there were two randomized controlled trials (RCTs), five longitudinal intervention studies, three systematic reviews, eight literature reviews, and one questionnaire survey. Among these, 15 articles addressed rehabilitation provided by nurses within a multidisciplinary rehabilitation, while four articles specifically discussed rehabilitation as one of the nursing interventions for brain tumor patients. Common future challenges noted in all articles included a lack of research on outcome measurement, insufficient knowledge and skills among individual nurses, and underdeveloped expertise in the field of rehabilitation nursing.

### Rehabilitation nursing for brain tumor patients

The rehabilitation nursing provided for brain tumor patients, as reported in the selected 19 articles, was categorized into five domains: information gathering and organization, giving meaning to continued rehabilitation, prevention and management of secondary complications, family support, and collaboration with other healthcare professionals.

**Table 1:** Abstract table

Author, Country	Title, Year of Publication	Aims	Study Design	Participants/sample size	Intervention	Outcome concept	Key Finding
YANG Wei-lin, China	A study on the intervention of nursing program integrated with rapid rehabilitation in perioperative patients with brain tumors, 2020	To study the effect of nursing program integrated with the concept of rapid rehabilitation on perioperative patients with brain tumors.	RCT	100 underwent surgery patients(50:50)	The control group’s nursing plan was routine nursing and the observation group made a nursing plan with the concept of rapid rehabilitation.	・QOL ・Complications	・Nursing intervention based on the concept of rapid rehabilitation in perioperative patients with brain tumors is of positive significance for optimizing surgical indicators, reducing complications and improving quality of life.
Hojan K & Gerreth K, Poland	Can Multidisciplinary Inpatient and Outpatient Rehabilitation Provide Sufficient Prevention of Disability in Patients with a Brain Tumor? -A Case-Series Report of Two Programs and A Prospective, Observational Clinical Trial, 2020	To evaluate the effectiveness of a multidisciplinary rehabilitation, carried out as an out- or in-patient program, as prevention of disability in BT patients.	Prospective, observational clinical study	Brain tumor patients who were allocated to inpatient (*n* = 28) or outpatient (*n* = 26)	Inpatients engaged in individual physical exercises for 150 min per day, six days a week, along with one hour of individual occupational therapy, neuropsychological therapy, and speech therapy for five days a week. Outpatients received exercise training for 120 min per day, five days a week, and had access to consultations with a psychologist, a social worker, and an occupational therapist.	・QOL ・Physical Function ・Cognitive Function	・The 12-week rehabilitation which started early after BT treatment as an inpatient or outpatient program had a positive role in improving functions in BT patients in all aspects of their functioning in terms of physical function, subjective neurocognition, and QoL. ・Comprehensive rehabilitation treatment to be implemented as soon as possible once oncological therapy is completed, for the purpose of preventing disabilities in this group of cancer patient.
Piil K et al., Denmark	Controlled rehabilitative and supportive care intervention trials in patients with high-grade gliomas and their caregivers: a systematic review, 2016	To give a detailed overview of non-pharmacological rehabilitative and supportive care interventions for patients with high-grade gliomas and/or their caregivers, and provide an appraisal of the methodological quality of these studies.	Systematic review	9 articles	Non-pharmacological rehabilitative and supportive care	・Physical Function ・Cognitive Function ・Psychological issues	・Cognitive group therapy improves memory skills in patients with high-grade gliomas, early physical training improves functional outcome and massage therapy reduces stress. ・Telephone follow-up and a specialist nurse function was an effective and useful way to achieve information and support.
ZHANG Juan et al., China	Effects of different swallowing training methods on postoperative dysphagia of patients with tumors in cerebellopontine angle region, 2020	To probe into nursing effects of different swallowing training methods on postoperative dysphagia of patients with tumors in cerebellopontine angle region.	RCT	60 dysphagia patients	Motor imagery training is imagine training while relaxing, and task-oriented training is programs tailored to patient challenges.	・Swallowing Function ・Complications	・Nurses guide patients through explanations and instructions so that they can actively participate in rehabilitation, aiming for the functions necessary in real life. ・Comprehensive interventions that combine and coordinate rehabilitation are effective.
Nordentoft S et al., Denmark	Evaluation of a multimodal rehabilitative palliative care program for patients with high- grade glioma and their family caregivers, 2022	To explore patient and caregiver experiences and evaluate the relevance of and satisfaction with a multimodal rehabilitative palliative care program for patients diagnosed with a high-grade glioma and their family caregivers.	Longitudinal multimethod study	17 high grade glioma patients and 16 their family caregiver	REHPA-HGG program offered participants new knowledge, counselling, physical training, and peer interactions.	・Program questionnaire	・Nurse was evaluated positively because she could establish personal relationships with family caregivers and provide ongoing personalized support. ・Intervention with nurses as a resource for family care
Al-Maqbali MA, Ireland	Glioblastoma multiforme in adults and the role of the advanced nurse practitioner, 2013	To explore a systematic approach to physical and psychosocial assessment through a description of the diagnosis, treatment and symptom management of glioblastoma multiforme.	Review	86 articles	-	-	・Nurses has a significant role in meeting the complex long-term needs of people with glioma. ・The key aspects of supportive care are communication, information, psychological and social support, and rehabilitation services. ・Many researchers have emphasized the importance of a multidisciplinary team approach, and nurses can play a key part in communicating with patients to identify their needs and refer them to specialists.
McCarty S et al., USA	Health-Related Quality of Life and Cancer-Related Symptoms During Interdisciplinary Outpatient Rehabilitation for Malignant Brain Tumor, 2017	To determine the relationships between functional outcomes, clinical symptoms, and health-related quality of life among patients with malignant brain tumors receiving interdisciplinary outpatient rehabilitation.	A nonrandomized, prospective, longitudinal study	49 adults with malignant brain tumors	A patient participated based on own needs in at least two of the three disciplines of therapy (PT, OT, and SLP), generally from 2 half days per wk (3 h/d) to 5 full days per wk (6 h/d).	・QOL ・Physical issues ・Psychological issues	・Interdisciplinary rehabilitation performed by a multidisciplinary team including nurses can prevent a decline in patients’ QOL and worsening of pain and depression.
Bartolo M et al., Italia	Improving neuro-oncological patients care: basic and practical concepts for nurse specialist in neuro-rehabilitation, 2012	To identify the standard competencies for neurorehabilitation nurses that could be taught by means of a specialization course.	Review and multi-choices questions while a semi-structured interview	147 articles	As educational method a structured course based on lectures and practical sessions	・Program questionnaire	・Standard competencies of the neurorehabilitation nurses: clinical, technical, methodological, organizational and legal. ・the development of rehabilitation depends on the improvement of scientific and practical knowledge of health care professionals, and this structured training course could be incorporated into undergraduate nursing education programs and also be inserted into continuing education programs for graduate nurses.
Khan F et al., Australia	Multidisciplinary rehabilitation after primary brain tumor treatment, 2013	To assess the effectiveness of multidisciplinary rehabilitation in adults after primary brain tumor treatment, especially the types of approaches that are effective (settings, intensity) and the outcomes that are affected.	Systematic review	1 article	Multidisciplinary rehabilitation is defined as any intervention delivered by two or more disciplines in conjunction with medical input to maximize activity and participation	・QOL ・Physical Function ・Physical issues ・Psychological issues ・Social issues ・Program questionnaire	・higher intensity ambulatory multidisciplinary rehabilitation reduces short- and long term disability(continence, mobility and locomotion, cognition) in people with brain tumor compared with standard outpatient care. ・multidisciplinary approach provides patients with skills needed to manage their own care. It prioritises patient-centred care and focuses on a person’s function and disability, using a goal-based.
Armenteros Borrell M et al.	Nursing research on the rehabilitation of neurological and neurosurgical patients, 1994	To highlight the role of nurses in the rehabilitation of brain tumor patients.	Review	6 articles	-	-	・Nurses begin rehabilitation as a process of the patient’s illness experience that begins immediately after diagnosis. ・Because decreased physical function is likely to inhibit psychological adaptation, nurses should be present to give the impression that they are interested in the patient and help the patient set goals for his or her future.
Andrejeva J & Volkova OV, Russia	Physical and Psychological Rehabilitation of Patients with Intracranial Glioma, 2018	To highlights the role of physical and psychological rehabilitation int the treatment of patients with intracranial glioma.	Review	75 articles	-	-	・Nurses is setting appropriate therapeutic goals with team. ・Engage so as not to withhold or delay efforts in the hope of a spontaneous recovery of sensation. ・Pain involves a prominent psychological component and approach social consequences, effective management requires multidisciplinary, including nurse.
Weyer-Jamora C et al., USA	Postacute Cognitive Rehabilitation for Adult Brain Tumor Patients, 2021	To review cognitive rehabilitation principles, discuss the evidence for cognitive rehabilitation, and provide guidance regarding the referral process.	Review	67 articles	-	-	・Cognitive rehabilitation is approached in (1)Acquisition, (2)Application, (3)Adaptation stages using the Triple A model. ・Acute and post-acute cognitive rehabilitation is effective in restoring patients’ cognitive functions related to attention, language, and executive function. ・Cognitive rehabilitation based on learning mechanisms synergizes with spontaneous and intervention-based recovery. ・Screening to consistently assess patients and subsequent intervention by multidisciplinary is effective. ・Methodological concerns, low rates of systematic cognitive screening, lack of awareness of cognitive rehabilitation in the brain tumor population, and rehabilitation provider scarcity remain barriers to care.
Pieczyńska A et al., Poland	Predictors of functional outcomes in adults with brain tumor undergoing rehabilitation treatment: a systematic review, 2022	To identify predictors of effective rehabilitation in aspects of physical functioning of BT patients.	Systematic review	21 articles	Rehabilitation aimed at improving physical function.	・QOL ・Physical Function	・Type of rehabilitation is home exercise program, proprioceptive neuromuscular facilitation (PNF) and Bobath method, and exercise program involved training the range of motion in the joints, strength training, improving balance and coordination, gait training, aerobic, and endurance training. ・The effectiveness of rehabilitation in improving physical fitness and coping with activities of daily living was confirmed. ・Rehabilitation of patients with BT can be multidisciplinary.
Spina S et al., Italia	Rehabilitation interventions for glioma patients: a mini-review, 2023	To classify and map rehabilitation interventions used to treat multiple disabling sequalae in individuals affected by glioma.	Review	13 articles	-	-	・This program featured to ensure adherence, such as allowing patients to exercise at home and providing regular guidance. ・Rehabilitation protocols typically include physical, occupational, psychological support, and cognitive therapies, and the timing of the rehabilitation can vary. The interventions included inpatient, outpatient, and home-based rehabilitation, with varying exercises and therapy components.
Vargo M et al., Sweden	Rehabilitation of patients with glioma, 2016	To delineate the long-term effects of rehabilitation interventions, and exploring impact of rehabilitation interventions on caregiver burden.	Review	160 article	-	-	・Nurses help to prevent complications that arise due to functional impairment and to maximize the effects of rehabilitation. ・Telephone support by nurses is highly valued by patients and families, and is also associated with rehabilitation effectiveness. The components were accessibility, positive information, and dedication.
Kirshblum S et al., USA	Rehabilitation of persons with central nervous system tumors, 2001	To review some of the basic aspects of rehabilitation for brain patients.	Review	58 articles	-	-	・Comprehensive rehabilitation can contribute to preventing complications, maximizing function, and improving quality of life for patients. ・The nurse assesses the patient’s level of independence and the nurse can use compensatory techniques to reinforce accordingly.
Sierpowska J et al., Netherland	The Aftercare Survey: Assessment and intervention practices after brain tumor surgery in Europe, 2022	To describe representative (neuro) psychological practices implemented after brain surgery in Europe.	An online survey	38 European centers completed the survey.	-	-	・Nearly half of the respondents offer support programs to caregivers, and all teams recommend them. Treatments differed between those offered to individuals with low-grade glioma vs. those with high-grade glioma.
Zwinkels H, Netherland	The developing role of the neuro-oncology nurse: a Dutch perspective, 2008	To describe the role of specialist nurses in care, cure and research for patients with brain tumors	Review	12 articles	-	-	・Daily activities of the specialist nurse is to discuss and advise on clinical and psychosocial matters. ・Nurses discusses treatment, its side effects and how the treatment will be evaluated with the patients. ・Nurses support patients with patient education, symptom management, and follow-up.
Roberts PS et al., USA	The impact of inpatient rehabilitation on function and survival of newly diagnosed patients with glioblastoma, 2014	To examine the impact of an inpatient rehabilitation program on functional improvement and survival among patients with newly diagnosed glioblastoma multiforme (GBM) who underwent surgical resection of the brain tumor.	Retrospectively cohort	412 glioma patients	Inpatient Rehabilitation Program: in a minimum of 3 h of therapy 5 of 7 days per week, 3 h of therapy, and some screening.	・Physical Function ・Complications	・Patients newly diagnosed with GBM who received inpatient rehabilitation demonstrated improvements in functional status from admission to discharge, with the highest gain observed in mobility.

#### Information gathering and organization

There were 12 articles that found information gathering and organization as part of rehabilitation nursing for brain tumor patients. These articles discussed the comprehensive gathering of psychosocial information from patients and families, as well as the organization of information to set goals for patients and families. This was done to initiate focused rehabilitation early in the treatment process.

Yang and Weyer-Jamora et al. reported on the collection of information by nurses regarding patients’ physical and cognitive functions, as well as their knowledge about treatment [20, 21]. Additionally, three articles discussed the collection of information from the time of admission based on the patient’s independence and daily life, taking into account the home situation, length of hospital stay, and potential transfer destinations [22–24]. Four articles emphasized the importance of monitoring all patient-related data and communication to understand the true needs of patients, in order to meet their psychosocial needs [5, 25–27]. These elements all involved the collection of information from patients and families.

Additionally, in four articles, setting goals with patients based on information collected from patients and families were addressed [28–31]. Particularly, Andrejeva and Volkova emphasized the importance of understanding patient needs when setting appropriate goals [29]. These aspects involved the process of organizing information based on collected data, hence categorized under information gathering and organization. All the studies emphasized the importance of information in the process of gathering and organizing data, including the patient’s treatment plan, current functional status, post-discharge living arrangements, and family situation. It was shown that structuring this information contributes to the development of the patient’s rehabilitation nursing care plan and goal setting with a focus on discharge, ultimately serving as the foundation for the patient to achieve autonomy and independence.

#### Giving meaning to continued rehabilitation

Eight articles found that giving meaning to the continuation of rehabilitation as part of rehabilitation nursing for brain tumor patients. These articles discussed the significance of patients with brain tumors actively and effectively engaging in rehabilitation for themselves.

In three articles, explanations about the illness, treatment, and rehabilitation, along with educational involvement, were presented [5, 20, 32]. Khan et al. and Hojan and Gerreth reported that consulting with patients about rehabilitation was important for giving meaning to the process [28, 30]. Additionally, three articles discussed follow-up through telephone calls or home visits to maintain connections with healthcare providers and information [11, 27, 33]. Specifically, Sierpowska et al. emphasized the value of continuity in transitioning from hospital to home and the importance of maintaining connections with healthcare providers and information [27]. Zhang et al. explained that imagery training for exercise rehabilitation by therapists with patients was effective [24]. Furthermore, Khan et al. reported that developing rehabilitation nursing plans tailored to the patient’s situation made the rehabilitation service available 24/7 as part of daily life [28]. Since all these interventions were aimed at giving meaning to the patients and supporting the continuation of rehabilitation, they were categorized as providing meaning to the continuation of rehabilitation. These studies commonly emphasized that, rather than nurses taking the lead in implementing care plans, patient autonomy is crucial for achieving the goal of rebuilding their lives. Based on this understanding, the importance of educational and supportive care provided by nurses was highlighted, as it can play a significant role in sustaining rehabilitation efforts.

#### Prevention and management of secondary complications

Eight articles discussed prevention and management of secondary complications as part of rehabilitation nursing for brain tumor patients. Four articles addressed physical aspects, while four articles focused on psychological aspects of preventing and managing complications. Let me explain each of these categories.

Yang reported that environmental adjustments allowing for early postoperative rehabilitation were effective in preventing and alleviating postoperative complications [20]. Al-Maqbali and Vargo et al. noted the need to address complications such as nutritional and skin integrity issues, as well as changes in digestive function resulting from brain tumors and their treatments [26, 34]. Nurses were urged to pay attention to prevent further serious symptoms resulting from these complications [26, 34]. Additionally, Andrejeva and Volkova stated that pain associated with rehabilitation implementation included not only physical but also psychological elements [29]. They emphasized the need to intervene in the multifactorial nature of pain experienced by patients, as it may impact social aspects when rehabilitation is inhibited by such pain [29]. These four articles addressed prevention and management of secondary complications on the physical aspect.

Regarding psychological aspects of complications, two articles pointed out the impact of decreased physical function in brain tumor patients on psychological aspects [25, 35]. Additionally, three articles reported the need for support in psychological aspects [5, 25, 35]. Particularly, Armenteros et al. revealed the potential hindrance of future adaptation due to changes in physical image [25]. They emphasized the importance of nurses being present and showing interest in patients as specific nursing support [25]. Zwinkels reported that symptom management through nurses’ educational involvement contributed to overall care management [32]. These studies noted that secondary complications have a psychosocial impact, as they result in longer hospital stays for brain tumor patients and reduce their willingness to continue treatment. They also highlighted the importance of preventing complications caused by tumors and treatment, as well as early detection, timely intervention, and supporting the patient’s natural healing ability as key aspects of rehabilitation nursing.

#### Family support

Three articles addressed the management of families in rehabilitation nursing for brain tumor patients.

Vargo et al. discussed the identification of concerns held by families [34]. Nordentoft et al. reported that nurses could facilitate ongoing individual support through building personal relationships with families [33]. Sierpowska et al. emphasized the significance of families as essential entities for patients, advocating for inclusive support through group meetings, information exchanges with nurses, and meetings with psychologists [27]. Brain tumor patients experience both cognitive and physical impairments, making it essential to seek cooperation not only from the patient but also from their family. If the ultimate goal is hospital discharge, home recovery, and reintegration into society, establishing a long-term collaborative system with the family becomes a crucial factor in helping the patient develop a sustained exercise habit.

#### Collaboration with other healthcare professionals

The literature addressing interdisciplinary collaboration in rehabilitation nursing for brain tumor patients consisted of seven articles. These articles mainly focused on support for daily living and enhancing communication within multidisciplinary teams. In all the literature, the importance of intervention by teams comprising various professions, including nurses, was highlighted.

Zhang et al. reported on the incorporation of rehabilitation interventions conducted by therapists into daily activities as part of nurses’ comprehensive interventions [24]. In two articles, nurses emphasized the importance of supporting the recovery and maintenance of functional abilities, safety, comfort, and overall quality of life in performing daily activities [34, 36]. These were nursing supports focused on daily living within the context of interdisciplinary rehabilitation.

Armenteros et al. reported on the importance of collaboration and information sharing with other healthcare professionals as vital elements of nursing for patients’ holistic adaptation [25]. Additionally, three articles highlighted communication as a crucial aspect of rehabilitation nursing, emphasizing nurses’ role in enhancing communication within interdisciplinary collaboration, thereby bridging the gap between other professionals and patients [26, 31, 32]. Such interdisciplinary collaboration was noted to enhance the effectiveness of rehabilitation within comprehensive teams [26]. All studies highlighted that in multidisciplinary rehabilitation, nurses serve as coordinators, bridging various professions. This category was established because a key role of rehabilitation nursing is to integrate the therapy provided by rehabilitation professionals into the patient’s daily life. Additionally, by understanding and sharing detailed patient functions observed through daily activities, nurses refine and personalize the interdisciplinary care plan to meet the unique needs of each patient.

## The effectiveness of rehabilitation nursing for brain tumor patients

There were no studies specifically focused on rehabilitation nursing. Even so, all the literature reported the effectiveness of the interventions.

The concepts most frequently used for measuring the effectiveness of rehabilitation nursing for brain tumor patients were QOL and physical function. The scales used to measure QOL included World Health Organization Quality-of-Life Scale (WHOQOL-BREF), MOS 36-Item Short-Form Health Survey (SF-36), and Functional Assessment for Control of Trunk (FACT) [20, 28, 30, 35, 36]. Among them, FACT was the most commonly used scale, employed in four studiesfor measuring QOL [28, 30, 35, 36]. The scales used to measure physical function included Functional Independence Measure (FIM), Barthel Index (BI), Berg Balance Scale (BBS), Karnofsky Performance Status (KPS), Fugl-meyer Assessment (FMA), Cancer Rehabilitation Evaluation System-short form (CARES-SF), The six-minute walk test (6MWT), modified Ashworth scale (MAS), and Postural Assessment Scale for Stroke (PASS) [11, 23, 28, 30, 36]. FIM was utilized in all studies measuring physical function [11, 23, 28, 30, 36]. 

### Effectiveness of rehabilitation nursing by type of interventions

Among the papers obtained in this study, rehabilitation nursing interventions were classified into those focusing solely on the exercise aspect and those incorporating both knowledge and exercise aspects. For example, in studies emphasizing the exercise aspect, Yang et al. reported that implementing a nursing plan centered on rehabilitation—including early mobilization, basic movement support, and discharge support—led to a reduction in complications and an improvement in patients’ quality of life (QOL) due to a shortened hospital stay [20]. On the other hand, interventions addressing both knowledge and exercise aspects included educational interventions that encouraged patient initiative and face-to-face exercise programs designed to enable patients to continue rehabilitation at home without direct medical support [22, 23]. The specific intervention methods are summarized in Table 1.

However, as noted above, a variety of scales were used to measure the effects of these interventions, with no common metrics identified. As a result, categorization remains challenging at this stage.

## Summary of findings: characteristics and challenges

### Characteristics of the target literature

In the field of cancer rehabilitation, various guidelines have been established based on evidence. Specifically, risk management and exercise prescription during exercise therapy, as well as treatment methods based on the stage of cancer, are globally recommended [9, 37, 38]. These serve as guidelines for clinical practice, research, and education. In the field of brain tumors, since the publication of the world’s first awake surgery guidelines in 2012 [39], the importance of supportive therapy to improve functional prognosis and maintain quality of life in brain tumor patients has increased [40]. Thus, since the 2010s, there has been a growing societal demand for rehabilitation for cancer patients and brain tumor patients worldwide. Therefore, among the 19 articles included, it is understandable that 16 were published after 2010.

This study revealed that there is insufficient accumulation of research findings to guarantee the effectiveness of rehabilitation nursing [11, 28, 36]. This is believed to be due to the difficulty in recruiting participants due to the characteristics of brain tumor patients, as well as the lack of standardization in measures. Indeed, in this study, a variety of concepts were used to measure the effectiveness of rehabilitation nursing, ranging from functional assessments to subjective evaluations based on patients’ experiences. This diversity may be attributed to nurses providing support with a holistic perspective, focusing not only on improving physical function or disabilities but also on supporting patients comprehensively.

Furthermore, in this study, 2,729 articles were excluded during the screening process. This exclusion may be attributed to several factors related to brain tumors, such as their rarity [41] and higher incidence rates in childhood [42]. Additionally, the central roles of physicians and therapists in functional recovery, which is the primary goal of rehabilitation, are also considered influential [43]. Moreover, unlike other neurological disorders, the illness experience of brain tumor patients extends back to before diagnosis [3], and rehabilitation is often performed as supportive therapy to complement primary treatments [8, 44]. Therefore, the illness experience of brain tumor patients exhibits distinctive characteristics compared to other neurological disorders, contributing to the exclusion of many articles during the screening process.

### Characteristics of rehabilitation nursing for brain tumor patients

The characteristics of rehabilitation nursing for brain tumor patients identified in this study all involve support provided by nurses to maximize the effectiveness of rehabilitation conducted by therapists, with no direct mention of functional recovery. This is a significant difference from previous studies that described the role of nurses in rehabilitation as caregivers [45], and emphasized activities maintenance, promotion, and functional recovery [46]. Rehabilitation, as implied by its etymology, entails resurrection or recovery [47], making support for functional recovery and maintenance an essential component. The reason for the absence of this crucial aspect could be attributed to the challenges faced in the academic development of rehabilitation nursing for brain tumor patients and insufficient linkage between daily life support and rehabilitation. Additionally, the strong reflection of the preventive aspect of rehabilitation, which is not typically observed in neurological disorders, could have influenced the results. However, elements related to recovery and maintenance are fundamental principles of nursing [48]. Therefore, it can be inferred that interventions including elements of recovery and maintenance are provided as part of daily life support for brain tumor patients. Thus, while the five domains extracted in this study could serve as foundational material for structuring the content of rehabilitation nursing practices for brain tumor patients, they are likely incomplete at this stage. Further exploratory research is needed to elucidate the practice of rehabilitation nursing.

Furthermore, among the nursing practices extracted in this study, the domains of information gathering and organization, prevention and management of secondary complications, and collaboration with other disciplines particularly reflected the characteristics of rehabilitation nursing for brain tumor patients.

Firstly, in the domain of information gathering and organization, elements of understanding the patient’s condition and needs, and setting goals based on them, were extracted. The technique of “understanding” is considered characteristic of rehabilitation nursing for brain tumor patients. Brain tumor patients may experience a decline in spontaneity and language impairments as the disease progresses [49–51]. For cancer patients, communication through language remains possible until approximately 1 to 2 months before death [52]. Additionally, in the case of stroke patients, who represent a significant portion of neurological disorders, significant brain function impairments occurring after injury often show good recovery within about a month after onset [53]. Therefore, in cases of cancer patients without brain lesions or stroke patients, there are many opportunities to listen to the patient’s needs through language. Thus, the element of “understanding” the conditions and needs of brain tumor patients, which may make it difficult to clearly express their needs and desired lifestyle compared to rehabilitation nursing for patients with other conditions, was emphasized in numerous studies.

Mentions of characteristic symptoms that emerge in brain tumor patients, particularly on the psychological aspects, extracted in the prevention and management of complications, reflect the psychosocial issues faced by these patients. Symptoms appearing in brain tumor patients vary depending on the location of the tumor, treatment strategies, and stage of treatment [44, 54]. Additionally, brain tumor patients exhibit both aspects as cancer patients and as individuals with brain function impairments, resulting in the emergence of various psychological disorders such as depression, fatigue syndrome, and emotional instability [55, 56]. In particular, seizures become highly stressful experiences for patients and their families [57]. Therefore, patients and families need to continue rehabilitation as one means of coexisting with brain tumors while coping with various symptoms. It is considered one of the characteristics of rehabilitation nursing for brain tumor patients to prevent and manage diverse and distinctive symptoms from a holistic perspective, considering the patient comprehensively.

Multidisciplinary collaboration, a fundamental principle of rehabilitation [58, 59], was also extracted as one of the elements of rehabilitation nursing for brain tumor patients. It has been reported that nurses need to integrate rehabilitation into daily life situations to contribute effectively to rehabilitation [60], which is consistent with the findings of this study. However, in previous research, nurses were found to have insufficient awareness of their role and responsibilities in rehabilitation [61, 62], and it was noted that they may not fully understand the roles and functions of other team members [63]. Additionally, for brain tumor patients, individualized rehabilitation tailored to each patient’s needs is necessary to address diverse symptoms and illness experiences [44]. Therefore, efforts should be made to establish rehabilitation nursing as a scholarly discipline, particularly to enhance multidisciplinary collaboration. This is considered a characteristic of rehabilitation nursing for brain tumor patients.

### The effectiveness and challenges of rehabilitation nursing for brain tumor patients

The importance of rehabilitation nursing has been recognized, and discussions on its expertise are progressing. However, when focusing specifically on brain tumor patients, a challenge remains: relatively little discussion has been conducted on rehabilitation nursing that considers the characteristic symptoms and disease progression of these patients. All intervention studies and randomized controlled trials (RCTs) adopted in this study reported the effectiveness of rehabilitation nursing for brain tumor patients. However, these reports only mentioned rehabilitation provided by nurses within a multidisciplinary rehabilitation, without detailing the support provided by nurses. Furthermore, the evaluation methods for effectiveness and safety, along with data collection methods, are recognized as common challenges both domestically and internationally [64, 65]. It is necessary to address these challenges and integrate the effectiveness and safety of rehabilitation nursing for brain tumor patients. Given the lack of foundational evidence [64], there is a need to accumulate research findings to establish the field of rehabilitation nursing for brain tumor patients, while referencing existing high-quality evidence from other fields.

It has also become evident that there is a lack of knowledge and skills among individual nurses regarding rehabilitation nursing for brain tumor patients. Previous studies targeting nurses involved with brain tumor patients have reported difficulties in patient interactions [66], as well as high learning needs in supporting patients with unique illness experiences [67], which align with the findings of this study. While the five domains of rehabilitation nursing extracted in this study are abstract, they are believed to reflect the characteristics of brain tumor patients. Utilizing these as one of the foundational materials, it is necessary to explore the specificity of these domains and consider methods for nurses to acquire knowledge and skills.

These challenges can be attributed to the underdevelopment of specialization in rehabilitation nursing for brain tumor patients. Specialization involves the integration of knowledge and skills and the ability to play a unique role within interdisciplinary collaboration [68]. Understanding one’s own and others’ expertise within interdisciplinary teams is also highlighted in the ladder requirements for rehabilitation [69]. Even though rehabilitation nursing may be invisible [70], researchers have documented the growing importance of strengthening interdisciplinary collaboration [30, 31, 71]. There is a need to articulate insights from the perspective of how nurses can function and contribute within enhanced interdisciplinary collaboration, aiming to maximize the effectiveness of rehabilitation.

## Limitations of the study and future directions

Due to the utilization of CINAHL Plus and PubMed databases in this study, there are limitations regarding the comprehensiveness of the review. This study aimed to provide an overview of the research, and did not conduct a rigorous analysis of intervention effectiveness through evaluation of publication bias or meta-analysis. In addition, the scales used to measure the effects varied, highlighting the need for further research to enable a more rigorous analysis of the effectiveness of future interventions.

Despite the increasing social interest in the field of this study, the number of research reports, particularly intervention studies, remains limited. Therefore, it is necessary to accumulate further knowledge and consider the possibility of conducting integrative reviews in future research.

## Conclusion

A scoping review was conducted on literature addressing rehabilitation nursing for brain tumor patients, resulting in the inclusion of 19 articles. Nurses involved in caring for brain tumor patients play a crucial role in collecting and integrating information on patients’ needs, goals, and functions from patients and collaborating with other healthcare professionals, and in preventing and managing various symptoms arising from the disease and treatment. While direct findings of functional recovery were lacking, these aspects represent the support nurses can provide to maximize the effectiveness of rehabilitation. However, research focusing on the nursing aspects of rehabilitation for brain tumor patients is limited, and at present, there is no guaranteed effective support method. Further accumulation of knowledge is desired to establish evidence demonstrating the effectiveness of interventions in rehabilitation nursing for brain tumor patients.

## Data Availability

The datasets generated and/or analysed during the current study are available in the figshare repository, [10.6084/m9.figshare.25664301].

## Declarations

### Addendum

Some content of this paper was presented at the 27th East Asian Forum of Nursing Scholars.
